# Bleomycin-induced flagellate dermatitis after sclerotherapy for venous malformation: A case report

**DOI:** 10.1016/j.jdcr.2026.01.061

**Published:** 2026-02-17

**Authors:** Dajana Smoljan, Vedrana Bulat, Mislav Mokos, Reinhart Speeckaert, Slavko Gašparov

**Affiliations:** a Department of Dermatology and Venereology, Sestre Milosrdnice University Hospital Centre, Zagreb, Croatia; b University of Zagreb, School of Dental Medicine, Zagreb, Croatia; c Department of Dermatology, Ghent University Hospital, Ghent, Belgium; d Department of Pathology, School of Medicine, University of Zagreb, Merkur University Hospital, Zagreb, Croatia

**Keywords:** bleomycin, cutaneous eruption, drug eruption, flagellate dermatitis, hyperpigmentation, venous malformation

## Introduction

Bleomycin is a glycopeptide antibiotic composed mainly of bleomycin A2 and B2, produced by *Streptomyces verticillus*. It is used widely in oncology, including in testicular cancer, Hodgkin and non-Hodgkin lymphomas, and squamous cell carcinoma, and is also administered intralesionally for vascular malformations. Its therapeutic action relies on DNA binding and the induction of strand breaks that halt cellular proliferation.

Flagellate dermatitis is a recognized cutaneous adverse reaction to bleomycin and is characterized by linear pruritic streaks that may evolve from erythema to hyperpigmentation. Reported incidence varies considerably, with figures ranging from 8% to 66%.^1^ The condition has been attributed to several mechanisms, including direct cytotoxicity, trauma-related induction, and immune-driven pathways.^2^

We describe a case of flagellate dermatitis occurring within hours of a single intralesional dose of bleomycin administered during sclerotherapy for a venous malformation, highlighting that clinically significant reactions may arise outside high-dose oncology settings.

## Case report

A 28-year-old woman presented to our dermatology clinic with pruritic linear lesions on the trunk. One month earlier she had undergone elective sclerotherapy for a venous malformation of the right soleus muscle. During the procedure, she received 10,500 IU of bleomycin via intralesional injection under epidural anesthesia, a dose within commonly used per-session ranges for sclerotherapy (often capped at approximately 15,000 IU).

Approximately 2 hours after the procedure, she noticed erythematous whip-like streaks across the trunk, which darkened in the following days. Pruritus intensified, although she remained systemically well. She reported no further eruptions following the intial one. Intramuscular chloropyramine once daily for 4 days gave limited benefit.

**Fig 1:**
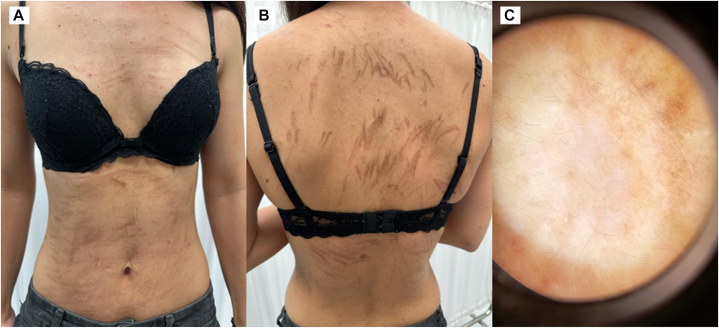
Clinical and dermoscopic features of bleomycin-induced flagellate dermatitis in our patient. **A,** The anterior trunk demonstrates multiple linear, whip-like *brownish* hyperpigmented streaks distributed across the chest and abdomen. **B,** The posterior trunk shows densely grouped, *brownish* flagellate streaks extending across the back. **C,** Dermoscopic examination reveals diffuse *brownish* pigmentation with irregular linear accentuation and background erythematous areas, corresponding to pigment incontinence and superficial dermal inflammation.

On examination, she had multiple brownish linear hyperpigmented streaks across both the anterior and posterior trunk (Fig 1). There was no mucosal involvement, no muscle weakness, and no systemic signs. She denied any recent ingestion of shiitake mushrooms and had no prior allergic, atopic, or urticarial history.

Her past medical history included volvulus surgery in childhood and a deep vein thrombosis in 2018. She was taking acetylsalicylic acid only. Immunologic evaluation, including antinuclear antibody, extractable nuclear antigen panel, myositis antibodies, anti-dsDNA, complement testing, rheumatoid factor, and serum protein electrophoresis, was normal. Chest radiography showed no abnormalities.

**Fig 2:**
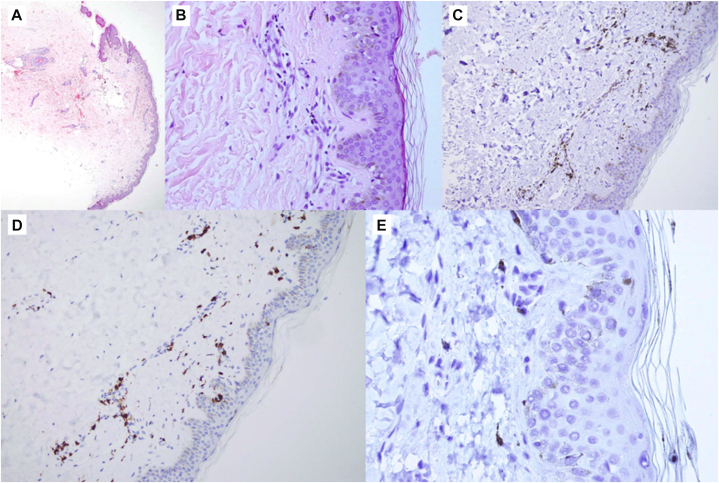
Histopathological and immunohistochemical findings of bleomycin-induced flagellate dermatitis. **A,** Hematoxylin and eosin staining (4×) shows focal epidermal thinning with a sparse superficial perivascular inflammatory infiltrate. **B,** Hematoxylin and eosin staining (40×) demonstrates scattered melanophages in the papillary dermis together with a mild lymphocytic infiltrate. **C,** Immunohistochemistry for CD4 (20×) reveals a predominance of CD4-positive T lymphocytes within the perivascular infiltrate. **D,** Immunohistochemistry for CD3 (20×) highlights the T-cell–predominant nature of the infiltrate in the superficial dermis. **E,** Immunohistochemistry for HMB-45 (60×) shows no evidence of melanocytic proliferation, confirming noncontributory staining.

A skin biopsy from the back was obtained for histopathological and immunohistochemical analysis, including hematoxylin–eosin, Masson’s trichrome, Giemsa, and staining for CD8, CD45, HMB-45, PAX-5, CD3, and CD4. Histologically, the epidermis was focally thinned, with scattered melanophages in the papillary dermis and a sparse perivascular infiltrate composed predominantly of T lymphocytes (CD3+, CD4+). Mild thickening of dermal collagen fibers was also noted (Fig 2).

Treatment consisted of topical betamethasone for 2 weeks, followed by a twice weekly application for another 2 weeks. Oral methylprednisolone 32 mg daily was prescribed for 7 days and then tapered by 4 mg weekly. At follow-up, partial improvement was observed, although persistent hyperpigmentation remained 2 months later.

## Discussion

Most cases of bleomycin-induced flagellate dermatitis occur in oncology, where cumulative exposure often exceeds 150,000 IU. Isolated cases at considerably lower doses have been documented, indicating that susceptibility varies. In our patient, the cutaneous reaction occurred following a single moderate dose of 10,500 IU of bleomycin, administered intralesionally for sclerotherapy of a benign venous malformation, outside of an oncologic context, suggesting that dose alone is not the sole determining factor.

The predominance of CD3+/CD4+ cells in the infiltrate suggests an immune-mediated contribution. Several mechanisms have been proposed, including direct injury to keratinocytes and endothelial cells, trauma-induced linear accentuation, and T-cell–mediated hypersensitivity.^2^^,^^3^ Clinical and histologic patterns across reports suggest that flagellate dermatitis represents a shared cutaneous response to varied pathways. Our findings support a dual mechanism involving immediate toxic effects and secondary immune activation.

The absence of other cytotoxic medications strengthens causal attribution. In this case, the eruption began approximately 2 hours after injection and then evolved in appearance (erythema to hyperpigmentation) without recurrent episodes, supporting a direct toxic effect or rapid local activation rather than a classical delayed-type hypersensitivity reaction. The presence of melanophages is consistent with injury to basal keratinocytes and melanocytes, leading to pigment incontinence and persistent hyperpigmentation.

Several differential diagnoses must be considered. Shiitake dermatitis is the closest mimic and results from exposure to lentinan, a constituent of *Lentinus edodes*, which triggers cytokine release, particularly interleukin (IL) 1, and can produce striking linear eruptions.^4^ Latency is usually 24 hours or longer, often accompanied by intense pruritus, and delayed skin test positivity has been described in some patients. In this case, there was no exposure, and the rapid onset after injection provides a clear alternative explanation.

Flagellate erythema in dermatomyositis is rare, with less than 5% of patients affected. Lesions often appear violaceous and may accompany systemic symptoms such as muscle weakness or interstitial lung disease.^5^^,^^6^ Serologic testing and clinical evaluation in our patient were normal, and lesion morphology was not typical for dermatomyositis. Adult-onset Still’s disease can produce linear eruptions linked to high levels of IL-1, IL-6, and IL-18, often in association with fever, arthralgia, and pulmonary involvement.^7^ None of these features were present here.

Several features of bleomycin pharmacology are relevant to understanding this reaction. The drug has a short plasma half-life and is rapidly metabolized by bleomycin hydrolase. Bleomycin demonstrates a degree of tissue selectivity, for example, skin and pulmonary tissue express very low levels of bleomycin hydrolase, an enzyme responsible for inactivating bleomycin, which binds metal ions in plasma, forming copper complexes that facilitate stability and uptake. In the cell, copper is replaced by iron, producing a bleomycin–iron complex capable of generating reactive oxygen species, which induce single-strand and double-strand DNA breaks.^8^^,^^9^ These properties explain both its antitumor action and its capacity for tissue toxicity. Low hydrolase activity in pulmonary tissue underlies the risk of pneumonitis and fibrosis, and a similar mechanism may contribute to cutaneous injury.^10^

Histologic features in this case, including epidermal thinning, pigment incontinence, and a CD4-dominant T-cell infiltrate, align with mixed cytotoxic and immune-mediated injury. The slow resolution of pigmentation reflects persistent dermal melanin deposition.

## Conclusion

This case illustrates flagellate dermatitis arising in a nononcologic context after a single intralesional dose of bleomycin. The rapid onset and absence of alternative triggers support a direct causal mechanism. Clinicians using bleomycin for sclerotherapy should be aware of this reaction to avoid unnecessary investigations and to counsel patients regarding expected recovery, including the possibility of prolonged hyperpigmentation.

## Conflicts of interest

None disclosed.

## References

[1] Indrastuti N., Mariyani S., Meidiyanti P. (2022). Flagellate dermatitis in bleomycin chemotherapy: a causality?. BMJ Case Rep.

[2] Cullingham K., Kost G. (2021). A case of bleomycin-induced flagellate dermatitis: a case report. SAGE Open Med Case Rep.

[3] Netchiporouk E., Pehr K., Ben-Shoshan M., Billick R.C., Sasseville D., Singer M. (2015). Pustular flagellate dermatitis after consumption of shiitake mushrooms. JAAD Case Rep.

[4] Nguyen A.H., Gonzaga M.I., Lim V.M., Adler M.J., Mitkov M.V., Cappel M.A. (2017). Clinical features of shiitake dermatitis: a systematic review. Int J Dermatol.

[5] Guimarães F., Teixeira F., Peixoto D., Teixeira V. (2021). Flagellate erythema: a rare cutaneous manifestation of dermatomyositis. Rheumatol (Oxford).

[6] Choi J., Lee J.J., Byun J.Y., Choi H.Y. (2025). Flagellate erythema: a rare presentation of idiopathic dermatomyositis. Indian J Dermatol Venereol Leprol.

[7] Toujani S., El Ouni A., Belhassen A., Bouslama K. (2022). Flagellate dermatitis: an atypical skin finding in adult-onset Still's disease. Clin Case Rep.

[8] Oppenheimer N.J., Chang C., Rodriguez L.O., Hecht S.M. (1981). Copper(I). bleomycin. a structurally unique oxidation-reduction active complex. J Biol Chem.

[9] Povirk L.F., Han Y.H., Steighner R.J. (1989). Structure of bleomycin-induced DNA double-strand breaks: predominance of blunt ends and single-base 5’ extensions. Biochemistry.

[10] Lazo J.S., Merrill W.W., Pham E.T., Lynch T.J., McCallister J.D., Ingbar D.H. (1984). Bleomycin hydrolase activity in pulmonary cells. J Pharmacol Exp Ther.

